# Ultrafast Interface Charge Separation in Carbon Nanodot–Nanotube Hybrids

**DOI:** 10.1021/acsami.1c16929

**Published:** 2021-10-05

**Authors:** Alice Sciortino, Francesco Ferrante, Gil Gonçalves, Gerard Tobias, Radian Popescu, Dagmar Gerthsen, Nicolò Mauro, Gaetano Giammona, Gianpiero Buscarino, Franco M. Gelardi, Simonpietro Agnello, Marco Cannas, Dario Duca, Fabrizio Messina

**Affiliations:** † Dipartimento di Fisica e Chimica—Emilio Segrè, Universitá degli studi di Palermo, Viale delle Scienze, Edificio 17, Palermo 90128, Italy; ‡ TEMA, Mechanical Engineering Department, University of Aveiro, 3810-193 Aveiro, Portugal; § Institut de Ciència de Materials de Barcelona (ICMAB-CSIC), Campus de la UAB, Bellaterra (Barcelona) 08193, Spain; ∥ Laboratory for Electron Microscopy, Karlsruhe Institute of Technology, Engesserstrasse 7, Karlsruhe 76131, Germany; ⊥ Dipartimento di Scienze e Tecnologie Biologiche, Chimiche e Farmaceutiche (STEBICEF), Università degli studi di Palermo, Via Archirafi 32, Palermo 90123, Italy; # CHAB—ATeN Center, Università degli studi di Palermo, Viale delle scienze, Edificio 18, Palermo 90128, Italy

**Keywords:** carbon nanodots, carbon nanotubes, ultrafast electron transfer, pump probe spectroscopy, carbon nanohybrids

## Abstract

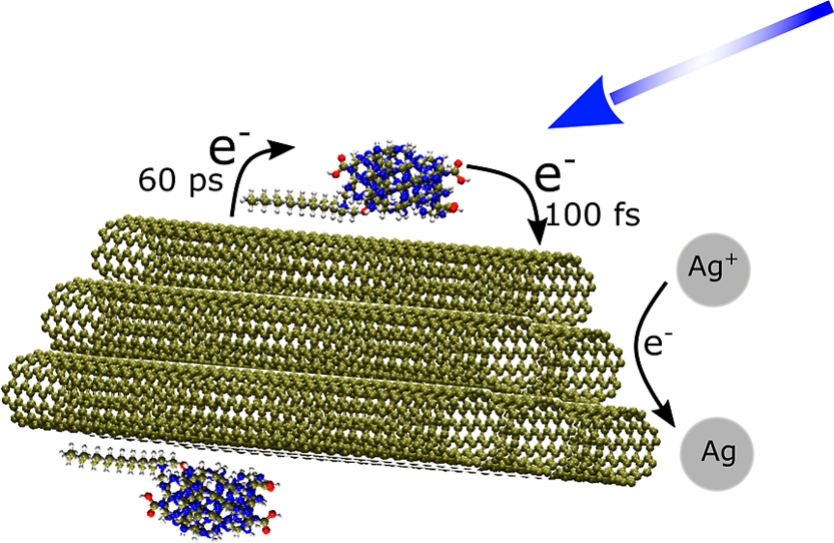

Carbon dots are an emerging family of zero-dimensional nanocarbons behaving as tunable light harvesters and photoactivated charge donors. Coupling them to carbon nanotubes, which are well-known electron acceptors with excellent charge transport capabilities, is very promising for several applications. Here, we first devised a route to achieve the stable electrostatic binding of carbon dots to multi- or single-walled carbon nanotubes, as confirmed by several experimental observations. The photoluminescence of carbon dots is strongly quenched when they contact either semiconductive or conductive nanotubes, indicating a strong electronic coupling to both. Theoretical simulations predict a favorable energy level alignment within these complexes, suggesting a photoinduced electron transfer from dots to nanotubes, which is a process of high functional interest. Femtosecond transient absorption confirms indeed an ultrafast (<100 fs) electron transfer independent of nanotubes being conductive or semiconductive in nature, followed by a much slower back electron transfer (≈60 ps) from the nanotube to the carbon dots. The high degree of charge separation and delocalization achieved in these nanohybrids entails significant photocatalytic properties, as we demonstrate by the reduction of silver ions in solution. The results are very promising in view of using these “all-carbon” nanohybrids as efficient light harvesters for applications in artificial photocatalysis and photosynthesis.

## Introduction

Increasing the performance of solar energy technologies and developing photocatalytic devices for efficient hydrogen production or for the removal of organic pollutants remain urgent scientific problems, in view of the key environmental issues involved. Today, nanotechnologies could provide new answers to these old problems. In particular, coupling nanomaterials with different electronic characteristics into a single device provides fundamentally new routes in materials science to develop efficient nano-photoelectrodevices for artificial photosynthesis, photocatalysis, and photovoltaics.^1−5^ Crucial requirements for these applications are the use of low cost materials and the possibility of sensitively controlling their characteristics to allow the fine tuning of the devices.^6^ Carbon-based nanomaterials are an area of huge interest in nanoscience motivated by their unsurpassed chemical versatility and diverse electronic properties which can be finely tailored for applications. Coupling carbon-based nanomaterials among them, or with other nanomaterials, holds great promise for the development of optically driven applications.^3,4,7−9^ In particular, the interactions between different nanocarbons are an area of intense investigation, pointing toward the perspective of an “all-carbon” nanotechnological paradigm.^7−9^

In this respect, one of the key problems is achieving a thorough fundamental understanding of the photophysics of carbon nanohybrids, which can be remarkably complex. Only the full comprehension of the electronic interactions between different nanocarbons would allow achieving the efficient operation of C-based nanocomposites in practical applications, which is often more difficult than anticipated on purely theoretical grounds. Here, following this idea, two carbon-based nanomaterials, carbon nanodots and carbon nanotubes, are electrostatically coupled and their photophysics is studied in detail.

Carbon nanodots (CDs) have emerged as a new frontier in nanoscience from the beginning of 2000s.^10^ CDs can be regarded as a rather broad family of carbon nanocolloids,^11^ which show up in a variety of slightly different forms. However, all of them typically display intense and tunable absorption–emission bands in the visible spectral range,^10,12^ which are their most important hallmark among other nanocarbons. Their excellent optical response is combined with many additional benefits such as low cost,^13^ biocompatibility,^14^ non-toxicity,^15^ and the great sensitivity to external agents as ions or molecules in solution.^16^ Most importantly, CDs also display remarkable electron-donating and electron-accepting capabilities.^7,16^ The combination of all of these characteristics makes CDs suitable substitutes of quantum dots in many optoelectronic or sensing applications.

Single-wall (SW) and multiwall (MW) carbon nanotubes (CNTs) are endowed with a range of exceptional properties ultimately related to their 1D structure.^17,18^ The properties of SWCNTs can be finely tailored based on their structure. Depending on chirality, their electronic characteristics can range from conductive to semiconductive.^19^ Moreover, by changing the diameter, one can tune the π-plasmon feature and the electronic band gap of semiconductive CNTs within the infrared spectral range.^20,21^ Decorating the surface of CNTs with various functional groups or molecules,^22^ it is also possible to increase their colloidal stability in different solvents and preparing them for coupling to other nanomaterials, such as CDs.

Here, we achieved the spontaneous electrostatic coupling of CDs to different types of CNTs and to explore the synergy between CDs as visible light absorbers with a high electron-transfer capability and CNTs as electron acceptors with good transport capabilities. Very few works have studied the photophysics of CD–CNT complexes so far,^23−25^ by covalently coupling CDs to CNT surfaces, while other studies have focused on CD–graphene^8^ or CD–graphene oxide hybrids.^26,27^ Overall, these works have shown a rather complex pattern of ground-state and excited-state interactions, highly dependent on the coupling pathway. Besides, achieving photoinduced charge separation, which lasts long enough to be directly translated into applications, has proven harder than expected, and the photocatalytic use of CD–CNT composites has not been addressed before.

Here, complexes of CDs with three different types of CNTs are investigated with a wide range of experimental techniques, including optical methods with temporal resolutions going from femtoseconds (fs) to the steady state, and computational studies to support the interpretation of experiments. We provide for the first time an upper limit of 100 fs for the timescale required for the excitation to migrate from the dots to either semiconducting or conductive nanotubes, a result which demonstrates an extremely efficient photoinduced charge separation at CD–CNT interfaces. This effect can be harnessed in photocatalytic applications as we demonstrate by efficient silver reduction. We also find that electron transfer is followed by a back-electron transfer (BET) to CDs in about 60 ps, which remains the main limiting factor to further exploit CD–CNT hybrids in applications. On the one hand, our results demonstrate the promise of CD–CNT nanohybrids as a new family of functional materials. On the other hand, we expect that these findings will pave the way for future developments in the field through an improved understanding of the fundamental photophysics of CD–CNT interactions.

## Materials and Methods

Carbon nanoparticles were synthesized as described hereafter. Conductive SWCNTs (c-SWCNTs) were supplied by Thomas Swan Co. Ltd. (UK) and purified as described below. MWCNTs and semiconductive SWCNTs (s-SWCNTs) with a nominal (7,6) chirality were purchased from Sigma-Aldrich. All other reagents and solvents were obtained by Sigma-Aldrich.

### Synthesis of CDs

Carbon nanodots have been synthesized as previously reported.^28^ 3 g of citric acid were added to 6 g of urea in dimethylformamide solution and heated to 160 °C for 4 h. The product has been washed by adding ethanol and by centrifugation. The collected dark powder was dispersed in water and then purified by a sephadex column chromatography. Extensive details on the synthesis are reported in ref (28).

### Purification of c-SWCNTs

Chemical vapor deposition grown Elicarb CNTs were supplied by Thomas Swan Co. Ltd. (UK). The as-received material contains SWCNTs and a fraction of DWCNTs, along with some impurities. Namely, amorphous carbon, graphitic particles, and catalytic iron nanoparticles coated with graphitic shells. The as-received material was ground in an agate mortar and pestle. The resulting fine powder was then loaded into a silica tube (4 cm in diameter that served as a sample holder), which was placed inside an alumina tube in the center of a tubular furnace. The system was completely purged with argon for 2 h to remove oxygen from the system. After that, CNTs were thermally treated with water steam at 900 °C for 4 h. This treatment removes the amorphous carbon present in the sample and graphitic shells that coat the catalytic nanoparticles, while preserving the tubular structure of the carbon nanotubes. The steam-treated CNTs were refluxed in 6 M HCl overnight in order to dissolve the catalytic inorganic particles that became exposed after the steam treatment. Then, the sample was extensively washed by filtration with distilled water until the pH of the filtrate was neutral. Finally, the sample was dried in an oven overnight at 100 °C.^29^ The amount of the inorganic residue present in the purified sample was determined by thermogravimetric analysis (TGA) using a NETZSCH instrument, model STA 449 F1 Jupiter. After the complete oxidation of the material under flowing air, the collected solid residue was measured to be 0.18 wt %. Taking into account that Fe (catalyst employed for the growth of the CNTs) will get oxidized to Fe_2_O_3_ during the TGA analysis, if we consider that all the residue is Fe_2_O_3_, a 0.18 wt % of Fe_2_O_3_ corresponds to a 0.13 wt % of Fe in the purified material (Figure S1). Scanning electron microscopy (SEM) analysis of the sample reveals the filamentous nature of the material. SEM was performed on a SEM Quanta FEI microscope. SEM images were acquired directly on the solid powder without dispersing the sample in any solvent, being the reason why the CNTs appear highly agglomerated (Figure S1).

### Steady-State Absorption

Absorption spectra have been recorded in a 1 cm quartz cuvette by a single beam optical fiber spectrophotometer (Avantes) in a spectral range of 200–1200 nm. All the measurements have been recorded at room temperature.

### Steady-State and Time-Resolved Nanosecond Emission

Steady-state fluorescence and emission decay kinetics were collected by the same setup in two different configurations. The emission was excited by a 5 ns tunable laser with 0.1–0.3 mJ/pulse and recorded by an intensified charge-coupled device camera which acquires emission spectra within a variable temporal window (gate width) with a controlled delay with respect to the laser pulse. The gate width was set at 1 ms or 500 ps to acquire steady-state emission spectra or time-resolved emission decay, respectively. In the latter case, the temporal resolution is about 0.2–0.3 ns.

### Transmission Electron Microscopy

High-resolution transmission electron microscopy (HRTEM) is carried out on an aberration-corrected FEI Titan3 80–300 microscope at 300 keV electron energy. Samples for HRTEM were prepared at room temperature in air by drop casting of a diluted suspension of carbon dot/CNT complexes in water onto an ultrathin amorphous carbon film (3 nm) on a holey carbon support film mounted on a 400 μm mesh Cu grid (Ted Pella Inc.). HRTEM images were evaluated by calculating their two-dimensional Fourier transform (FT), which yields information on the crystal structure (lattice parameters and crystal symmetry) of single carbon dot/CNT complexes. The analysis was performed by comparing experimental FTs and calculated diffraction patterns with Miller indices, where the latter were obtained by using Jems (Java version of the electron microscopy simulation) software.^30^ On FT images, the zero-order beam is indicated by a white circle.

### Raman Spectroscopy

Raman experiments were performed exciting at 633 nm (1.96 eV) using a LabRam HR-Evolution spectrometer (HORIBA, Europe) equipped with a confocal optical microscopy system with 100x optical magnification, the best spectral resolution equal to 7 cm^–1^ and a data pitch equal to 1 cm^–1^. All the measurements were performed at a nominal power equal to 4 mW on a target area of 1 μm^2^.

### ζ-potential

The measurements have been performed on 1 mL of the fresh sample at room temperature using a Malvern Zetasizer NanoZS instrument equipped with a 632 nm laser with a fixed scattering angle of 173°, and Dispersion Technology Software 7.02 software (Malvern Panalytical ltd, Almelo, The Netherlands). The zeta-potential values (mV) were calculated from electrophoretic mobility using the Smoluchowski relationship. All analyses were performed in triplicate.

### Transient Absorption Spectroscopy

TA measurements were based on a 5 kHz Ti:sapphire femtosecond amplifier (Spectra Physics Solstice-Ace), which produces 75 fs pulses peaking at 800 nm with a full width at half-maximum of 30 nm at 350 mJ per pulse. 80% of the power is used to generate the pump and 20% generates the probe. The probe (400–750 nm) is generated focusing the 800 nm beam in a 1 mm quartz cuvette containing D_2_O. The probe is focused on the sample by a 150 mm parabolic mirror. The pump passes through a homemade non-collinear optical parametric amplifier to generate tunable wavelength (from 495 to 570 nm with a bandwidth of 10–20 nm). After the generation of the chosen wavelength, the pump is chopped at a repetition rate of 2500 Hz and then focused on the sample. The pump–probe delay is controlled by a motorized delay stage. The probe and the pump spatially overlap inside the sample, which continuously flows in a 200 mm thick flow cell upon the action of a pump, in order to hit with every pump pulse a fresh portion of the sample. After the sample, the probe beam is dispersed through a silica prism and focused on the detector by a lens. The spectral resolution is 3 nm. The pump and probe are synchronized using a single-shot camera detector system (Glaz Linescan-I) with 1024 pixels. A typical signal is obtained by averaging 5000 pumped and 5000 unpumped spectra for each delay, and scanning over the pump–probe delay 40–50 times. The measurements were carried out under magic angle detection conditions. The data presented in the paper were subjected to standard correction procedures, which eliminate the effects of cross-phase modulation and group velocity dispersion. The temporal resolution is about 70 fs.

The kinetics are fitted by a multiexponential function as ..., and the average lifetime is estimated as follows: .

### Atomic Force Microscopy

A drop of aqueous solution of silver nanoparticles was deposited on a mica substrate and then dried in vacuum. Atomic force microscopy (AFM) measurements were carried out in air using a Bruker FAST-SCAN microscope equipped with a closed-loop scanner (*X*, *Y*, *Z*) maximum scan regions: 35, 35, and 3 mm, respectively. Scans were obtained in the soft tapping mode using a FAST-SCAN-A probe with an apical radius of about 5 nm, and each image was obtained with a resolution comparable to the tip radius.

### Computational Details

All the computational data reported in this work are the results of calculations performed by means of the full third order self-consistent-charge density functional tight binding method^31^ as implemented in the DFBT+ program,^32^ joined with the 3OB set of Slater–Koster parameters^33^ and the following value: −0.1857 (H), −0.1492 (C), −0.1535 (N), and −0.1575 (O), for the derivative of the Hubbard parameters, with damping exponent equal to 4.0.

## Results and Discussion

**Figure 1:**
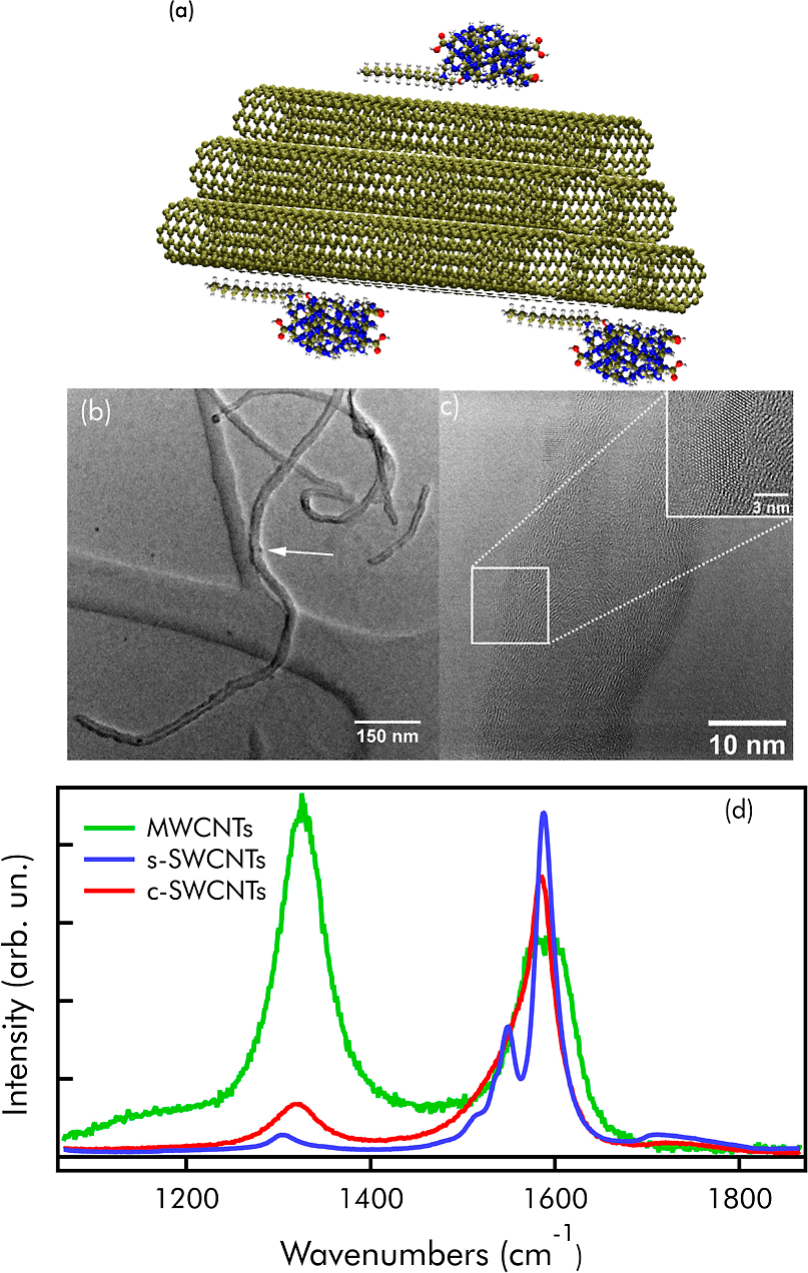
(a) Model of a CD-SWCNT complex held together by electrostatic interaction mediated by a CTAB molecule. (b) TEM image of the complex formed by CDs and MWCNTs. The white arrow indicates a single CD binding to the wall of nanotube (c) HRTEM image of the complex. The white square indicates a single CD adhering to the nanotube. A zoom of the square is shown in the inset of the figure, (d) normalized Raman spectra of MWCNTs (green line), semiconductive s-SWCNTs (blue line), and conductive c-SWCNTs (red line) excited at 633 nm.

To investigate the interaction between CDs and various types of CNTs, we designed the experiment to induce their spontaneous coupling through an electrostatic route. The CDs we used are described in detail in a previous publication^28^ and in the Methods and Materials section. They are small (<4 nm) fluorescent nanoparticles with a carbon nitride core structure (Figures 1b and S2) and a negatively charged surface (ζ-potential ≈ −20 mV). They are obtained by the solvothermal decomposition of citric acid and urea, a reaction which can be described as polycondensation ultimately resulting in the formation of nanocrystalline cores which are more or less densely covered at the surface by carboxylic and amide groups arising from the starting precursors. The degree of the surface coverage is critical for the optical properties as these functional groups are directly involved in the optical transitions of CDs^34^

Then, we selected three different types of CNTs: commercial multiwalled CNTs (MWCNTs) with internal and external diameters of 10 and 20 nm, respectively, and two different types of single-walled CNTs of commercial origin, here called s-SWCNTs and c-SWCNTs. The latter was purified in-house by steam treatment.^35^ All types of CNTs are hydrophobic and have negligible aqueous dispersability. For this reason, we used cetyl trimethyl ammonium bromide (CTAB) as a surfactant to force their dispersion in water: suspensions of 0.05 mM of MWCNTs or SWCNTs were typically prepared by dispersing the nanotubes in a 0.2 mM CTAB solution and sonicating in a bath for ∼1 h. Then, the supernatants containing isolated CNTs coupled with CTAB molecules were collected and then mixed at room temperature with CDs in aqueous solution. After mixing, samples were typically allowed to equilibrate for 30 min before measurements. The final concentration of CDs after mixing is 10 mg/L. To evaluate the effect of CD–CNT interactions, these samples were compared with solutions of bare CDs, which were equally prepared at a concentration of 10 mg/L for the sake of consistency.

Molecular dynamics^36^ predicts that CTAB molecules should spread along the long body of the nanotubes (Figure 1a), in order to maximize mutual hydrophobic interactions. While the positively charged polar tail of CTAB interacts with water allowing the dispersion of the nanotubes, it also allows the coupling of CNTs to CDs, acting as an electrostatic linker. In fact, the polar tail of CTAB remains available to electrostatically link to the surface of CDs, as favored by their strong negative charge.^28^ Thus, when a CNT–CTAB suspension is mixed with CDs, nanotubes and carbon dots should spontaneously get in close contact and form a stable complex, as pictured in Figure 1a. Of course, a certain degree of bundling between CNTs in solution cannot be excluded, so that only a portion of CNTs are expected to actually couple to CDs (see Figure 1a).

Later on in the paper, we provide proof of this experimental hypothesis by spectroscopic analysis of the samples. Nevertheless, to directly visualize CD-CNT complex formation, the mixed solutions were also analyzed by HRTEM (Figure 1b,c and S3). As expected, HRTEM images and their Fourier transforms (Figure S3) confirm the observation of CNTs in close contact to the surface of CDs. Similar results are obtained when CDs are allowed to interact with s-SWCNTs, c-SWCNTs, or MWCNTs (Figure 1b,c and S3). The nanohybrid solution is stable within a month.

The interaction between CDs and CNTs is expected to depend on the electronic properties of CNTs, controlled by their structure.^20,37^ Thus, the electronic properties were addressed by Raman analysis on the powder of the three types of CNT samples (Figure 1d). The Raman spectrum of MWCNTs shows a G-band peaking at 1591 cm^–1^ and the ratio between G and D-band intensities suggests a high grade of disorder of the system. The inspection of the Raman spectra in the G-band region (≈1600 cm^–1^) shows that the G-band of commercial s-SWCNTs splits into a high- and a low-frequency components, which is a well-known^37,38^ feature of semiconducting nanotubes. Indeed, the semiconducting character of these CNTs is consistent with their nominal (7,6) chirality.^37^ The samples contain some double-walled CNTs as demonstrated by HRTEM (Figure S4). In contrast, the G-band of c-SWCNTs (Figure 1d) is not split but appears asymmetrical with a tail at lower wavenumbers, indicating^37^ that c-SWCNTs are conductive.

Once established the conductive or semiconductive nature of the nanotubes, we focused our attention on CD-SWCNT interactions, by performing optical measurements with different time resolutions, further supported by computational investigations.

**Figure 2:**
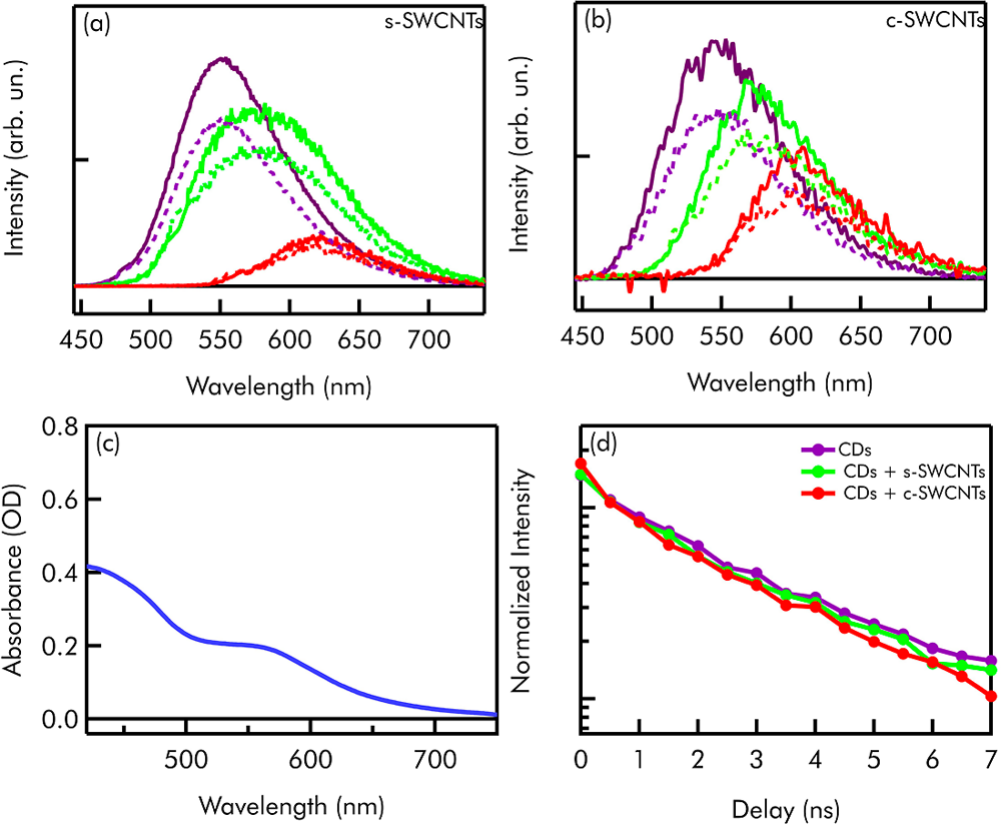
(a) Emission spectra of CDs (continuous lines) and of CDs + s-SWCNTs (dashed lines) excited at 480 nm (purple line), 520 nm (green line), and 560 nm (red line). (b) Emission spectra of CDs (continuous lines) and of CDs + c-SWCNTs (dashed lines) excited at 480 nm (purple line), 520 nm (green line), and 560 nm (red line). (c) Absorption spectrum of bare CDs. (d) Decay kinetics of the fluorescence of bare CDs (purple line), CDs + s-SWCNTs (green line), and CDs + c-SWCNTs (red line) excited at 540 nm. All the samples are prepared in 0.2 mM CTAB. The concentration of CDs is 10 mg/L in all the samples.

Figure 2c shows the optical absorption (OA) of CDs in water. Here, we focused on the lowest energy transition at λ > 480 nm. Exciting this band produces an emission whose peak position ranges from 550 to 650 nm depending on the excitation wavelength (see Figure 2). The electronic transition of these CDs has been recently investigated,^34^ finding that photoexcitation can be described as an electron transfer from nitrogen atoms inside the core to charge trap states localized on −COOH surface groups.

Figure 2a,b displays emission spectra of CDs (continuous lines) at different excitation wavelengths, prepared in 0.2 mM of CTAB because we found that CTAB addition in itself leads to a modest increase of the emissive quantum yield (not shown). These spectra are compared with the emission spectra of their complexes with s-SWCNTs (Figure 2a) and c-SWCNTs (Figure 2b) prepared as described above. To make the intensity comparisons meaningful, both bare CD and mixed CD solutions were prepared in such a way to ensure that the final concentrations of CDs and CTAB are always 10 mg/L and 0.2 mM, respectively, in all the spectra displayed in Figure 2. No precipitation of the samples is observed upon mixing. Therefore, the changes observed in the data are exclusively due to the addition of CNTs, thus reflecting the effect of CD-CNT interactions. Figure 2 shows that the emission of CDs is quenched in the presence of SWCNTs of both semiconducting and conductive characters. The degree of quenching is found to be around 30–35%. Similar results have been obtained when CDs are interacting with MWCNTs (Figure S5). The presence of CTAB slightly increases the emission of CDs, probably because it screens the CD from the solvent. On the contrary, close contact with CNTs causes a quenching of emission. Similar experiments have been conducted trying to couple CDs and CNTs through the action of sodium dodecyl sulfate (SDS) which has a negative polar tail. The coupling has not been possible with SDS and no quenching has been recorded (not shown). This suggests that the coupling with CTAB is electrostatic and no π–π stacking coupling between CDs and CNTs occurs as previously observed.^39^ Interestingly, the coupling is quite stable considering that the emission quenching is perfectly reproducible for at least an entire month. Time-resolved measurements have been used to further investigate the nature of this coupling. Nanosecond-resolved fluorescence kinetics in the absence and in the presence of SWCNTs, as shown in Figure 2d, are almost perfectly overlapping. The emission lifetime is 3.5 ± 0.5 ns. This demonstrates that the quenching of the emission is non-collisional and must take place in a sub-nanosecond temporal range, within stable CD-SWCNT complexes held together electrostatically by CTAB. Nevertheless, no evidence of significant ground-state interactions is found here, appearing for instance as a change of the steady-state OA of CDs upon coupling. In this sense, the present results partially disagree with previously reported evidence from ref (24), where, in addition to a photoinduced electron transfer, strong ground-state electronic interactions were observed as well. Here, in the close-contact arrangement between CDs and CNTs demonstrated by HRTEM, and considering the high density of acceptor states on CNTs, one would expect such a photoinduced electron transfer to be highly efficient and to occur on very fast timescales.

In order to learn more about the electronic properties of CD-SWCNT complexes, we performed density functional tight binding calculations (DFTB; see the Materials and Methods section for computational details), focusing on a model of CD/s-SWCNT.

With the aim of representing the most relevant structural features of CDs that are directly connected to their optical response, the model employed for the carbon dot is a particle cut out of bulk β-C_3_N_4_ and saturated with hydrogen atoms on the surface; it has C_65_N_81_H_79_ stoichiometry and a maximum size of about 1.2 nm. The surface of the particle was functionalized with four carboxylic groups and a carboxylate group; the latter interacts with a completely extended CTAB molecule, CH_3_(CH_2_)_15_N(CH_3_)_3_^+^. In the optimized geometry, the ion pair has a maximum size of about 3.8 nm, and the distances between the N atom of the CTAB and the O atoms of the carboxylate are 3.55 and 3.34 Å. The HOMO level of the CTAB-CD system is at −3.94 eV and the lowest unoccupied molecular orbital (LUMO) at −0.98 eV; the first is localized on the central nitrogen atom of the CD particle and the second is a π* orbital in one of the carboxylic groups.

The repeating unit of SWCNT(7,6) consists of 508 atoms and was built by using the TUBEGEN program.^40^ Periodic calculation on SWCNT(7,6) give a band gap of 0.79 eV, which is in perfect agreement with the analytical estimates on the dependence of the CNT band gap on chirality reported in ref (41). In the present work, a non-periodic model of SWCNT(7,6) was used, which is formed by two repetition units and characterized by a total length of about 10 nm. Considering the size of the CTAB–CD adduct, this SWCNT model is long enough to correctly describe the interactions in the complex and its electronic structure. The end parts of the SWCNT were saturated with hydrogen atoms; this causes the appearance of spurious electronic levels within the small band gap. These levels are localized just in the two terminal parts, so they were precisely identified and were not taken into account in the discussion. Therefore, excluding the states above, the HOMO level in the non-periodic model is at −4.79 eV and the LUMO at −3.94 eV, with a band gap of 0.85 eV, slightly greater than the one calculated for the periodic system.

**Figure 3:**
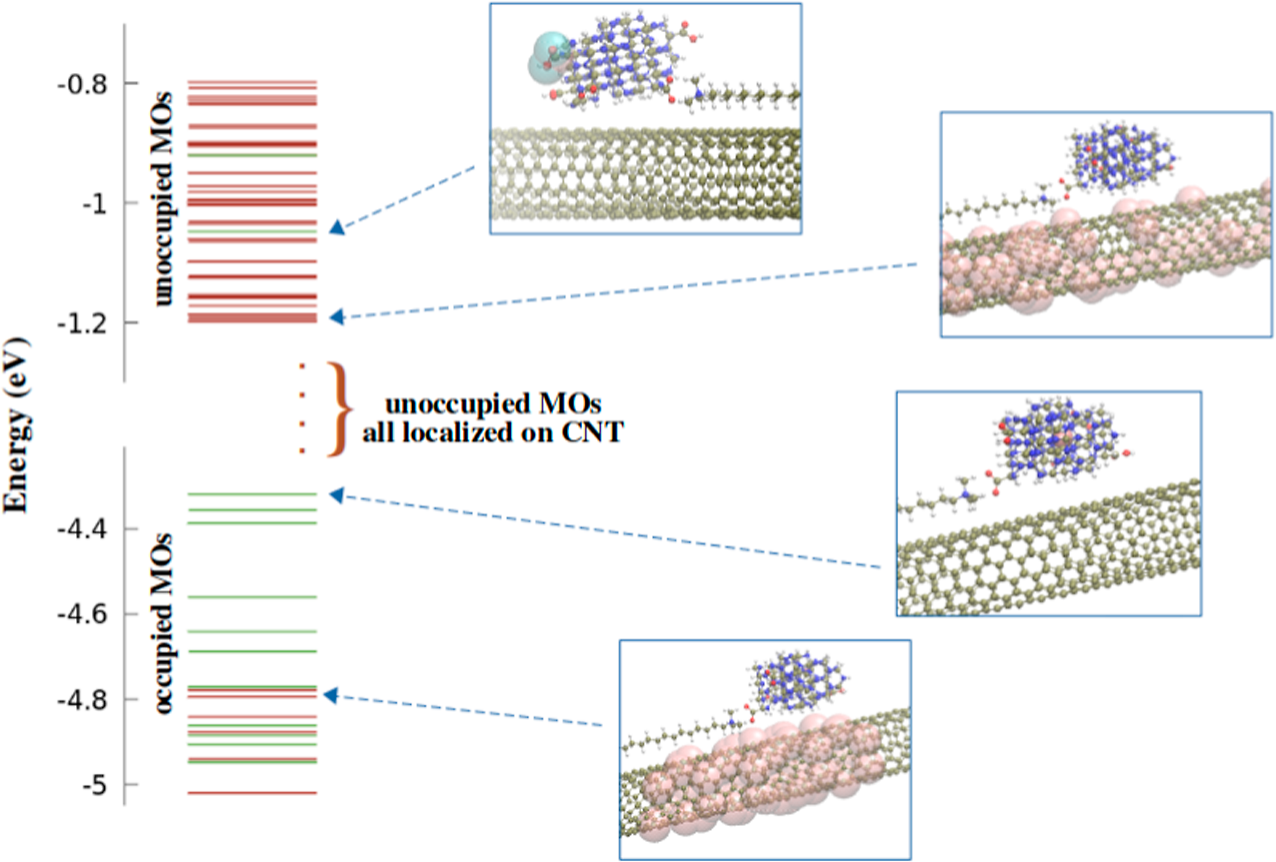
Molecular orbital (MO) energy levels of the CD-CTAB-SWCNT(7,6) complex calculated by a density functional tight binding approach. Molecular orbitals completely delocalized on the SWCNT are represented by red lines, those localized on CD are represented by green lines. On the right, some representative schematization of the MOs, comprising the highest occupied molecular orbital (HOMO) and the first unoccupied state localized on CD is shown.

In the complex of Figure 1a, CTAB and CD are electrostatically coupled, and they interact with SWCNTs through weak van der Waals forces. By considering the closest distances between the H atoms of the CTAB alkyl chain and the C atoms of the nanotube surface, in the optimized geometry of the complex, the CTAB chain is located at a distance of 2.7 ± 0.1 Å from the surface. The DFTB method estimates an interaction energy between CTAB-CD and SWCNT(7,6) as low as 15 kJ mol^–1^ (0.15 eV). Accordingly, we expect that the energy associated with the molecular orbitals of the complex does not change drastically if compared to the isolated counterparts. As a matter of fact, the HOMO is at −4.26 eV and is completely localized on CD, while the LUMO, at −3.85 eV, is instead delocalized on the central portion of the SWCNT surface. The first unoccupied orbital which is localized on the CD (the π* on one of the carboxyl groups) is at energy −1.02 eV. Within an interval of ±0.2 eV with respect to this state, that is, between −0.80 and −1.20 eV, there are 36 states all delocalized on the SWCNT(7,6) (see Figure 3). Therefore, although of qualitative nature, the calculations demonstrate the possibility that an electronic transition occurring on the CD may result in a charge transfer on the SWCNT, explaining the sub-nanosecond fluorescence quenching inferred from Figure 2.

To investigate the dynamics of the complexes on a faster timescale and look for direct evidence of the undergoing electron transfer, we performed transient absorption (TA) measurements by pumping the samples with 70 fs laser pulses tuned at 550 nm. In particular, here we probe the signal in the visible range in order to investigate the CD dynamics in the absence and in the presence of nanotubes. This approach is complementary to ref (24) where they probed the TA signals of CNTs in the NIR range.

In Figure S6, the typical TA signal of CDs (500 mg/L + 0.02 mM of CTAB) is shown. The signal is dominated by a broad negative contribution around the excitation wavelength, which is already present at the earliest time delays, and can be ascribed to a combination of the so-called ground-state bleaching (GSB) and stimulated emission (SE) signals.^42^ We also see an excited-state absorption signal at λ < 500 nm, probably due to the transition to bi-exciton states.^42^ Both negative and positive TA components are observed to decay in the explored temporal range (200 ps), although not completely, with an average lifetime of 3 ps. The signal is comparable to the TA signal of bare CDs (not shown) indicating that CTAB molecules do not influence the dynamics.

These TA experiments were then repeated on CD + s-SWCNTs and CD + c-SWCNTs mixed dispersions, prepared in such a way that the concentrations of CDs and CTAB are identical (500 mg/L and 0.02 mM) to the ones used in the TA experiments on bare CDs. Broadly speaking, we find that the TA signal of CDs after the addition of CNTs is similar to the one in Figure S6. However, several significant variations are observed. First, the intensity of the signal collected from the complexes is strongly reduced with both SWCNT types (example in Figure S7, top panel), with some changes of the shape in the TA signal, and both these changes are already observed at the earliest time delays accessible by our experiment (example in Figure S7, bottom panel). This result defines an upper limit for the timescale of electron transfer, which must have already occurred within our temporal resolution (∼ 100 fs) causing the dramatic change in signal intensity from the earliest time delay. Admittedly, a more specific attribution of the changes of the TA signal observed upon coupling CDs to CNTs is not straight forward, especially in the absence of other evidence such as, for example, one could be obtained in principle by spectroelectrochemical measurements. For example, we cannot entirely rule out that the observed changes are not only due to electron transfer but also due to a fast, concurrent energy transfer from the dot to the nanotubes. However, considering the high electron donor capabilities of CDs and their very close proximity to the nanotubes, we speculate that a fast electron transfer is more probable than any other option. In fact, this idea is strongly supported not only by the results of Figures 2 and 3 but also by the photocatalytical behavior of these composites, as described in the last part of the paper.

Such a remarkably high efficiency of the electron transfer is in line with our expectations and promising for applications. Indeed, a similarly ultrafast formation of a charge-separated state was found in other types of complexes involving CDs,^3,43^ confirming the high efficiency of CDs as photoexcited electron donors. However, this behavior is not ubiquitous in CD photochemistry. For example, other findings^26^ suggested a relatively slow electron transfer from CDs to graphene oxide. In a more focused parallel, a recent study on covalently bonded CD–CNT complexes observed an electron transfer over a comparatively much slower time constant of 300 ps.^23^ This difference suggests that in the design of our nanohybrids, electrostatic interactions allow much closer range interactions between CDs and CNTs, highly enhancing the coupling and promoting the formation of a CD^+^/SWCNT^–^ charge-separated state. Our data do not allow us to highlight the possible role played by CNT–CNT bundling in the electron-transfer process. Because our time resolution does not allow one to discriminate processes whose total duration remains less than 100 fs, it is possible for example that the primary electron-transfer event from CDs and CNTs may be followed by intertube charge transfer, contributing to the increase in the separation between the electron and hole. Nevertheless, in the following discussion, we consider only the simplest scenario, that is, the occurrence of a simple ultrafast electron transfer from the CD surface to CNTs.

**Figure 4:**
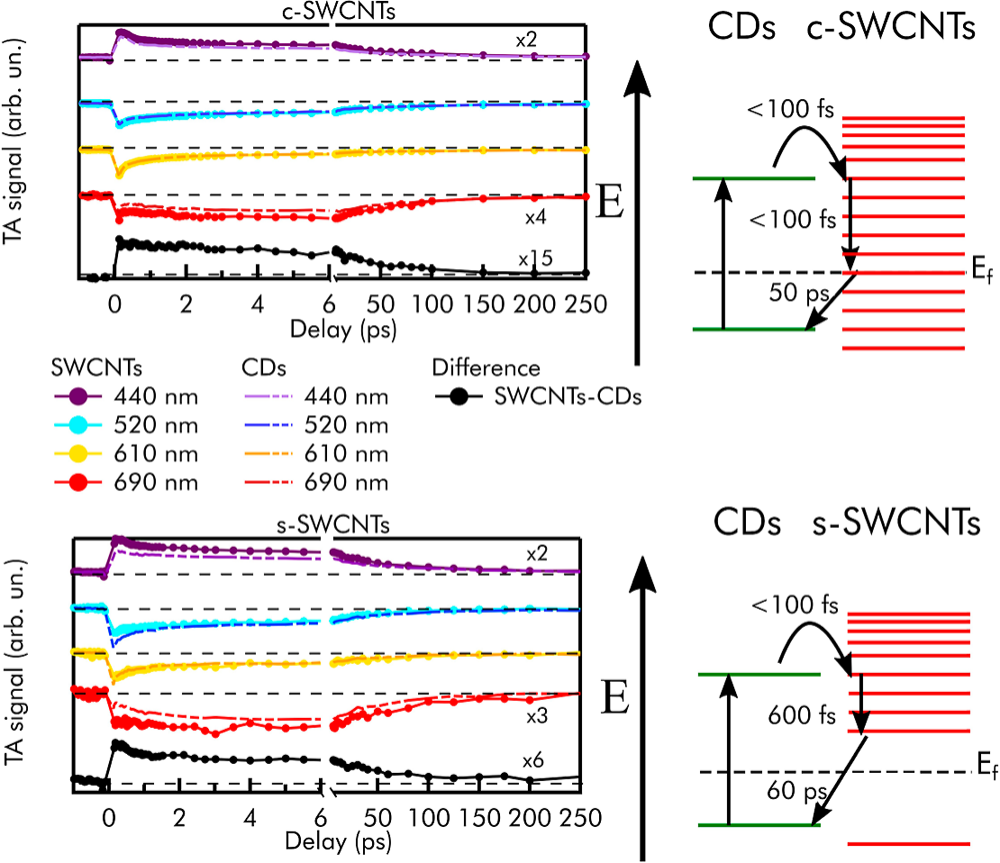
Transient absorption kinetics at 440 nm (purple line), 520 nm (light blue line), 610 nm (yellow line), and 690 nm (red line) of bare CDs and CDs + c-SWCNTs (top panel) and bare CDs and CDs + s-SWCNTs (bottom panel). Difference kinetics calculated at 440 nm between CD-CNT complexes and bare dot ones (black curves in both panels). Schematized models of forward- and back-electron transfer from CDs to SWCNTs and back, with the related characteristic timescales as obtained by a fitting procedure. In all of these measurements, the samples were prepared in order to ensure a fixed concentration of CDs equal to 500 mg/L.

It is worth noting that no TA signal is detected from the pure SWCNT dispersion because of their very low concentration. Therefore, the observed differences between the isolated CDs and CDs in the presence of SWCNTs are entirely due to the mutual interactions. In this respect, the dynamical evolution of the CD^+^/SWCNT^–^ state can be isolated by comparing the TA dynamics of bare CDs with CD-CNT complexes. A meaningful way to do this comparison is normalizing the TA signals of bare CDs and CD-CNT complexes in the spectral region of the SE. This normalization (see Figure S7) is justified because even for the CD-CNT sample, the SE signal only arises from the non-quenched CDs (i.e., a minor portion of CDs which are not bound to CNTs). Once the signals were normalized in this way, several kinetic traces at different probe wavelengths were extracted and compared to each other, as shown in Figure 4.

We find that the TA kinetics recorded at 610 nm probe wavelengths are practically identical in bare CDs and CD-CNT complexes, confirming that the signal in this region is mostly due to the SE of non-complexed CDs. This is almost exactly observed also for the TA kinetics at 520 nm, which is dominated by the GSB of non-complexed CDs. On the other hand, the kinetics recorded at 440 and 690 nm probe wavelengths reveal differences between bare CDs and CD-CNT complexes. As can be seen from Figure 4, these differences are maximized at short time delays, and tend to disappear at long time delays. These findings are found to be reproducible in repeated experiments.

Then, in order to isolate the dynamics of the complexes, we calculated the difference between the kinetics at 440 nm of bare CDs and CD-CNT complexes, obtaining the black curves in Figure 4. These data can be considered as a fingerprint of the dynamics of the CD^+^/CNT^–^ complexes after the electron transfer has occurred. When CDs are coupled to conductive c-SWCNTs, these difference kinetics decay with a timescale of ∼ 50 ps, as extracted from a fitting procedure (Figure S8). When CDs are coupled to semiconductive s-SWCNTs, the decay kinetics has a bi-exponential behavior with characteristic times of ∼0.6 ps and ∼60 ps (Figure S8). The fact that these difference kinetics ultimately decay to zero means that the photocycle of the complexes, initiated by ultrafast electron transfer, is completed at longer times by a BET from CNTs to the CDs. Therefore, we can interpret 50 ps in the conductive case and the 60 ps in the semiconductive case as the characteristic times of the BET from CNTs to CDs. The additional 0.6 ps timescale we find in s-SWCNTs is consistent with studies on bare SWCNTs^44,45^ and probably represents an internal relaxation of the complex before BET. A similar BET dynamics (80 ps) is recorded in the CD-MWCNT hybrids (not shown).

In a sense, the occurrence of BET is not surprising. Because of electrostatic attraction, the electrons injected in the CNT will ultimately tend to recombine with the hole left on CDs, a process which should tend to limit the lifetime of the electron–hole pair. Despite this, the average lifetime of the excited state is substantially prolonged to 50–60 ps with respect to bare CDs, where we find an average decay lifetime of 3 ps, as already mentioned above. Notably, the picosecond recombination of the charge-separated state was recently proposed in two previous studies focusing on a different type of the CD-CNT complex.^24,46^ However, no evidence of ultrafast electron transfer was discussed in these studies.^24^

Based on these results, and considering the alignment of the energy levels, the dynamics of the CD-SWCNT nanohybrids is finally modeled as depicted in Figure 4. The photoexcitation of CDs produces an almost instantaneous electron transfer from the surface of the dot to the CNTs (100 fs), forming a CDs^+^/SWCNT^–^ charge-separated state at the interface. A fast electron transfer such as the one recorded here strongly suggests the very close contact between CDs and CNTs, only separated by the short surface functional groups on CD surfaces. In particular, if any organic molecules (e.g., impurities occasionally observed in the bottom-up CD synthesis) were placed between the donor and the acceptor, then a much longer timescale should be expected. Immediately after that, an internal relaxation drives the optically active electron to the bottom of the conduction band of CNTs. Such internal relaxation in nanotubes is known to be very fast:^44,45^ based on our data, we propose a characteristic timescale of 0.6 ps for s-SWCNTs, whereas in the case of c-SWCNTs, it is so fast that we cannot reveal it. A faster internal relaxation in the conductive CNTs is indeed consistent with the higher density of levels expected in the conduction band. After the relaxation, the electron finally undergoes a BET to CDs which closes the photocycle. Based on our analysis, the BET occurs on slightly different timescales of 50 ps and 60 ps for c-SWCNTs and s-SWCNTs, respectively.

The 50–60 ps lifetime of the charge-separated CD^+^/SWCNT^–^ state should suffice to behave as a photoactivated charge donor for further photochemical processes. This point is particularly interesting considering that the photocatalytic function of CD-CNT nanohybrids has not been previously addressed in the existing literature.

**Figure 5:**
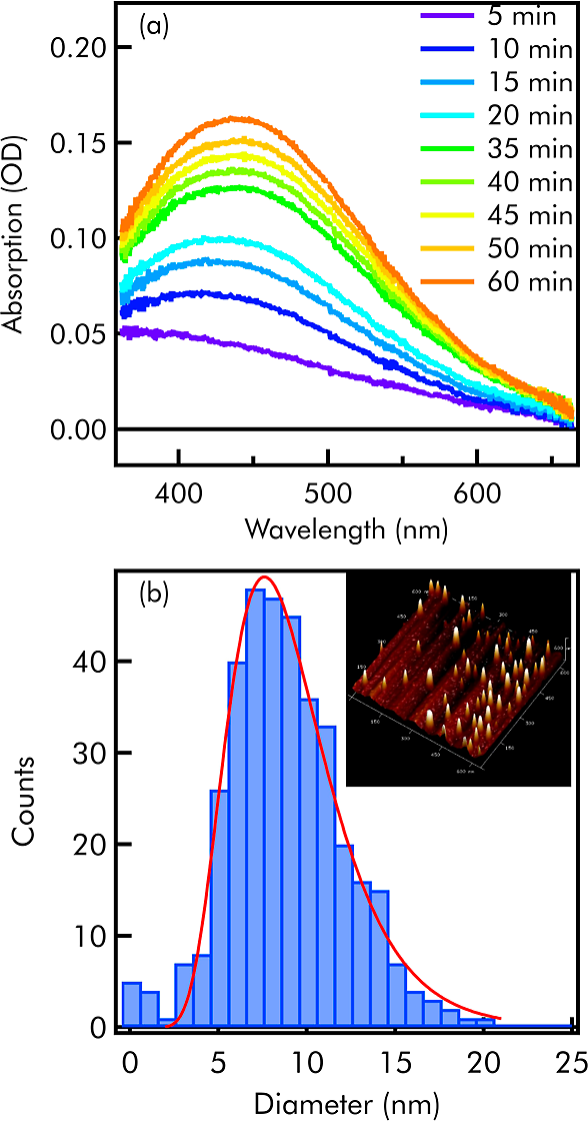
(a)Absorption spectra of silver nanoparticles synthesized from the light exposure of CD^+^/SWCNT^–^ complexes recorded at different exposure times. (b) Size distribution of silver nanoparticles (from CD^+^/SWCNT^–^) after 1 h of exposure.

To show this, we carried out a proof-of-principle experiment aimed at demonstrating the photocatalytic use of the nanohybrids in the synthesis of Ag nanoparticles (NPs). We added 400 μM of Ag_2_SO_4_ and 5% of 2-propanol used as a hole scavenger to aqueous solution of CD + s-SWCNT and exposed it to the visible light from a gas discharge lamp. The complete redox process photocatalyzed by our system is therefore the reduction of silver accompanied by the oxidation of 2-propanol. As shown in Figure 5a, in these conditions we observe the growth of the plasmonic absorption at 430 nm of Ag NPs in a matter of minutes. Atomic force microscopy confirms the formation of quasi-spherically Ag NPs with an average diameter of 8 nm (Figure 5b). From the estimation of the molar extinction coefficient of 8 nm sized Ag NPs, we calculate a concentration of 600 pM nanoparticles formed after 1 h of exposure. A scheme of the photocatalytic process is reported in Figure S10. No Ag NPs are formed without light exposure. No Ag NPs are formed if the Ag_2_SO_4_ solution is illuminated in the presence of CNTs only. In contrast, also the isolated CDs are capable of functioning as light-activated electron donors (Figure S9a). However, in this case, we get a very different result: the AgNPs formed upon light exposure show substantially larger and highly variable sizes (10–40 nm) and shapes, their plasmonic absorption band is much broader and strongly redshifted (510 nm), and their concentration in solution after 1 h of exposure (estimated from OA) is 10–50 times lower than found with CD-CNT nanosystems.

On the one hand, despite the excited state of bare CDs having a short average lifetime (3 ps, from Figure S6), their ability to reduce Ag should not be surprising considering the substantial core-surface charge-transfer character of their excited states.^34^ In contrast, silver reduction by CD–CNT nanohybrids proceeds through photoinduced charge transfer from their longer-lived (50 ps) CD^+^/SWCNT^–^ excited states. In this sense, both bare CD and CD-CNT systems behave as photocatalytically active nanomaterials displaying different properties. In particular, the ability of CD-CNT hybrids to photocatalyze the formation of small Ag NPs with better-controlled shapes and high efficiency is consistent with the starting hypothesis of this study, and very promising for further developments. Thanks to the delocalization of the charge over the nanotubes, the interface-separated CD^+^/SWCNT^–^ state features a highly enhanced charge delocalization as compared to bare CDs. Therefore, the CD-CNT nanocomplexes provide a much larger active area where Ag^+^ reduction can occur, and thus many more nucleation sites can be initiated, ultimately leading to the growth of a large number of small NPs of a better-controlled shape. In addition, the longer average lifetime of the charge pairs in the nanohybrid as compared to bare CDs further contributes to more efficient photocatalysis.

## Conclusions

We have explored a route to assemble stable CD/CNT complexes and addressed their photophysics by the combined use of several different techniques. The electrostatic coupling of carbon dots and carbon nanotubes produces a quenching of CD emission which stems from a very efficient electron transfer occurring in less than 100 fs from the photoexcited dot to the coupled nanotube. The occurrence of this process is predicted by theoretical simulations and consistent with the results of femtosecond TA measurements. Different from some previous studies, the two nanosystems do not appear to undergo significant ground-state charge-transfer interactions, the electron transfer from CDs to CNTs being only triggered by photoexcitation. The electron transfer is followed by a back electron transfer from the CNT to the CD with a lifetime of 50–60 ps, which depends on the electronic properties of the nanotube. The increased lifetime and strongly enhanced charge separation and delocalization in the photoexcited CD-CNT nanohybrids intensify their photocatalytic activity as compared to bare CDs. Our results are very promising in view of the functional use of CD/CNT complexes in artificial photocatalytic or photosynthesis applications and provide clear clues on how the performance could be further enhanced. We anticipate that further studies should pursue a suitable chemical engineering of the CD–CNT interface directed at further prolonging the lifetime and spatial separation of the charge-separated state, by preventing back-electron transfer, to the point of achieving an irreversible charge separation across CD–CNT interfaces.

## Supporting Information Available

HRTEM, SEM, and AFM images of CNTs and CDs, optical characterization by steady-state fluorescence, and TA (PDF)
