# Supplementary material for: Ultrafast Interface Charge Separation in Carbon Nanodot–Nanotube Hybrids
Source: ACS Appl Mater Interfaces. 2021 Oct 5;13(41):49232–41. doi: 10.1021/acsami.1c16929 (PMC8532113; doi:10.1021/acsami.1c16929)
Supplement: Supplementary file 1 — am1c16929_si_001.pdf [file am1c16929_si_001.pdf]

Supporting Information

Ultrafast interface charge separation in carbon nanodot-nanotube hybrids

Alice Sciortino,*,† Francesco Ferrante,† Gil Gonçalves,‡ Gerard Tobias,¶ Radian Popescu,§ Dagmar Gerthsen,§ Nicolò Mauro,∥ Gaetano Giammona,∥ Gianpiero Buscarino,†,⊥ Franco M. Gelardi,† Simonpietro Agnello,†,⊥ Marco Cannas,† Dario Duca,† and Fabrizio Messina*,†,⊥

†*Dipartimento di Fisica e Chimica - Emilio Segrè, Universitá degli studi di Palermo, Viale delle Scienze, Edificio 17, 90128. Palermo, Italy*

‡*TEMA, Mechanical Engineering Department, University of Aveiro, 3810-193 Aveiro, Portugal*

¶*Institut de Ciència de Materials de Barcelona (ICMAB-CSIC), Campus de la UAB, 08193 Bellaterra (Barcelona), Spain*

§*Laboratory for electron Microscopy, Karlsruhe Institute of Technology, Engesserstrasse 7,76131, Karlsruhe, Germany*

∥*Dipartimento di Scienze e Tecnologie Biologiche, Chimiche e Farmaceutiche (STEBICEF), Università degli studi di Palermo, Via Archirafi 32, 90123, Palermo, Italy*

⊥*CHAB - ATeN Center, Università degli studi di Palermo, viale delle scienze, Edificio 18, 90128, Palermo, Italy*

E-mail: alice.sciortino02@unipa.it; fabrizio.messina@unipa.it

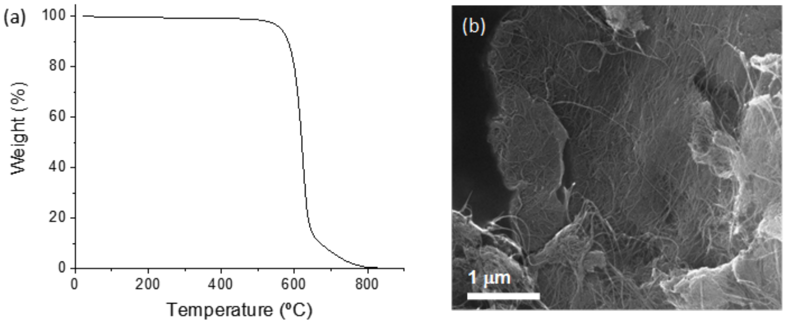

Figure S1: Characterization of the purified c-SWCNTs. a) TGA analysis underflowing air at a heating rate of 10º C/min;b) SEM image of the material.

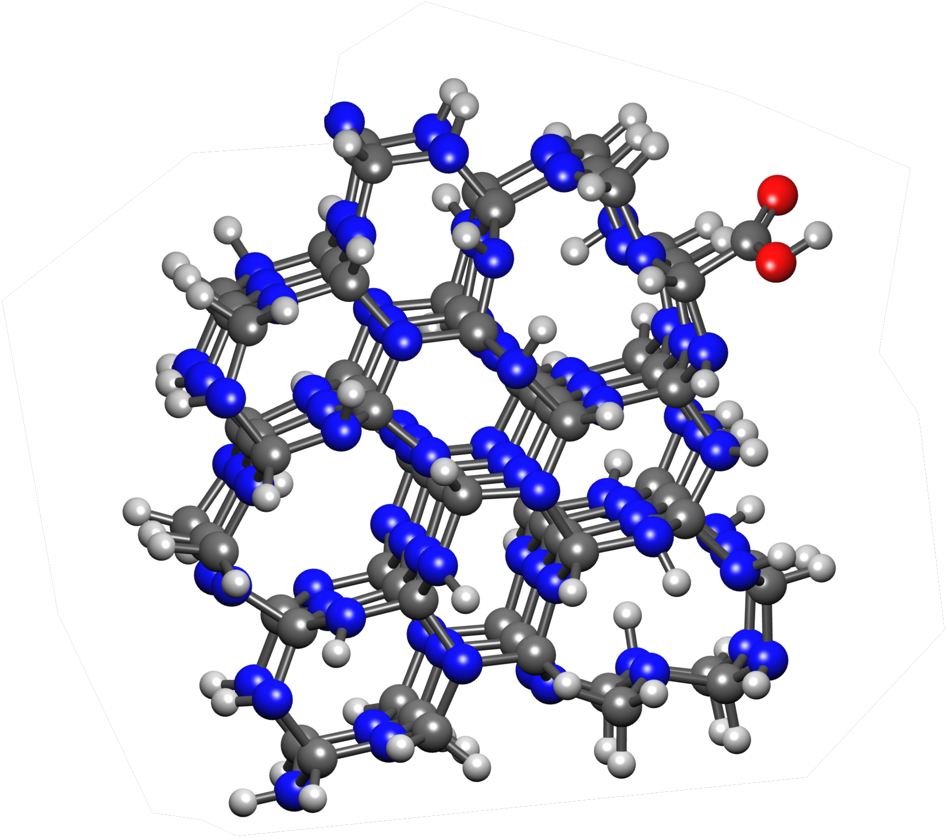

Figure S2: Structural model of Carbon dot core with a carboxylic group attached on the surface.

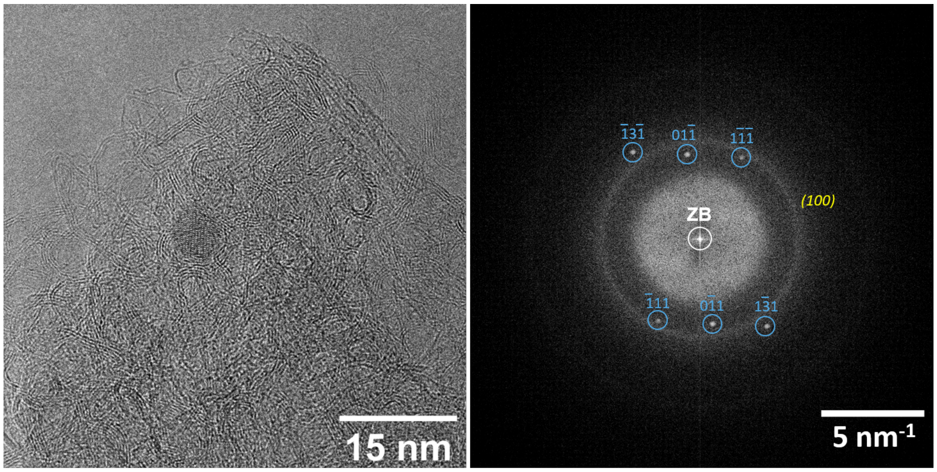

Figure S3: HRTEM image of the complex formed by CDs and conductive nanotubes (c-SWCNTs), with the corresponding Fourier transform (FT). The FT displays contribution from (i) a single monocrystalline carbon dot with a slight distorted $\beta - C_3N_4$ hexagonal structure (space group P63/m, space group number 176) in the [211]-zone axis (blue symbols and Miller indices) and (ii) the Debye-Scherrer ring belonging to SWCNTs, which is attributed to the (100)-reflection of the bulk hexagonal C-graphite (space group P63/mmc, space group number 194 (yellow Miller index)

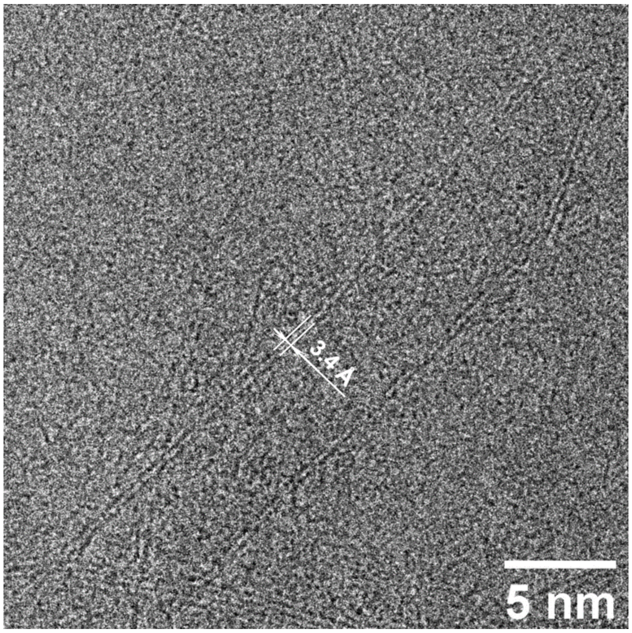

Figure S4: HRTEM image of a single DWCNT of s-SWCNTs sample.

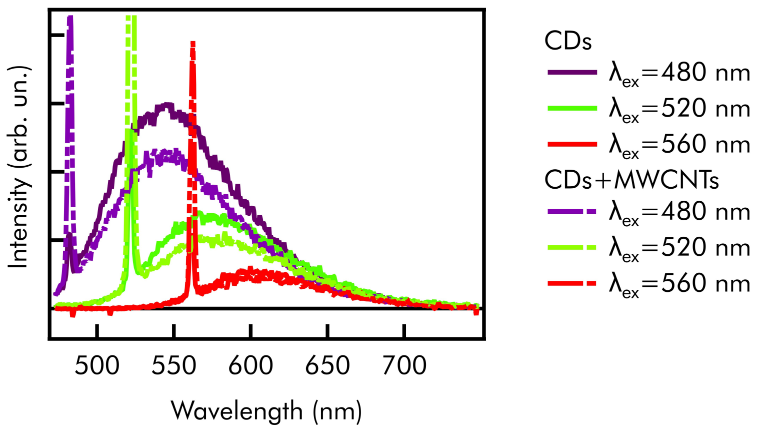

Figure S5: Emission spectra of Cds (continuous lines) and of CDs+MWCNTs (dashed lines) excited at 480 nm (purple lines), 520 nm (green lines) and 560 nm (red lines).

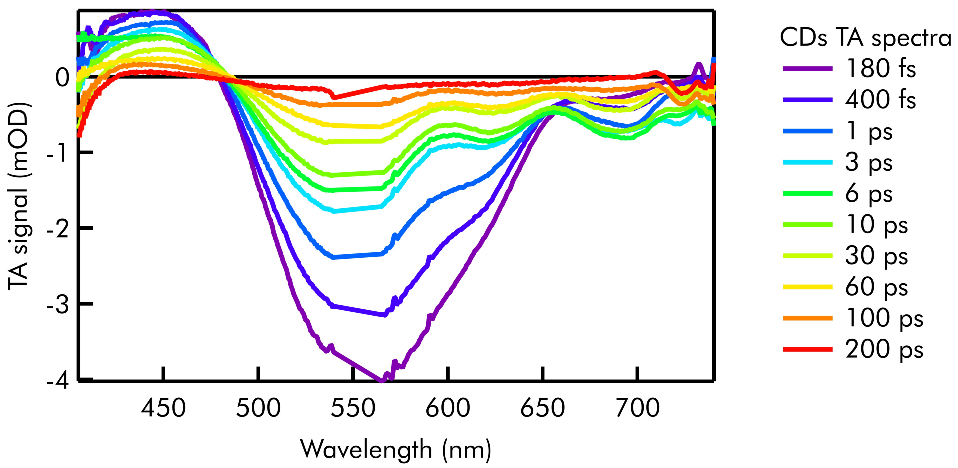

Figure S6: Transient absorption spectra of CDs solution, excited at 550 nm, as a function of delay.

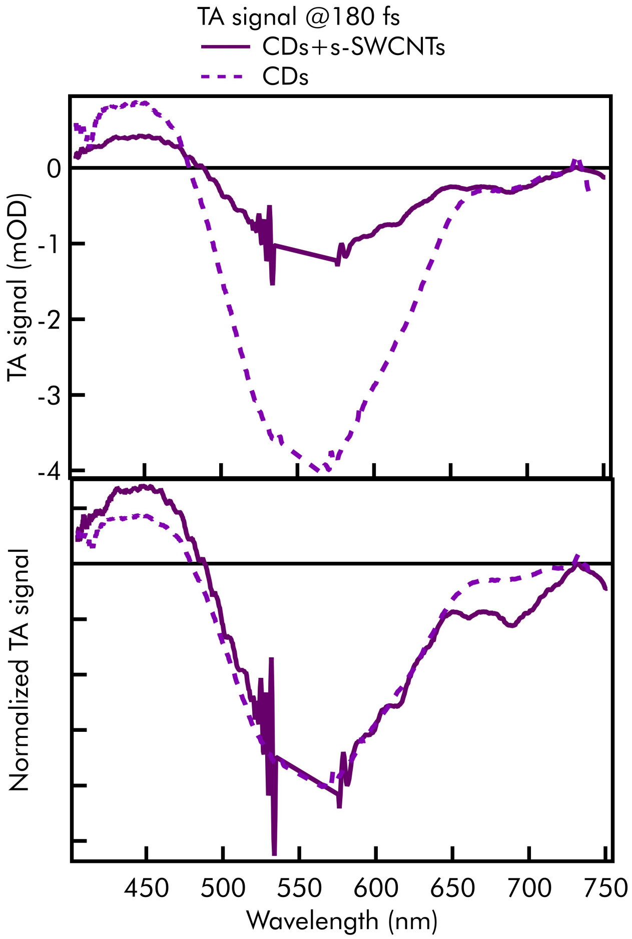

Figure S7: Top panel: Comparison between TA signals of CDs and CDs + s-SWCNTs recorded at 180 fs. Bottom panel: Normalized TA spectra of CDs and CDs + s-SWCNTs at 180 fs.

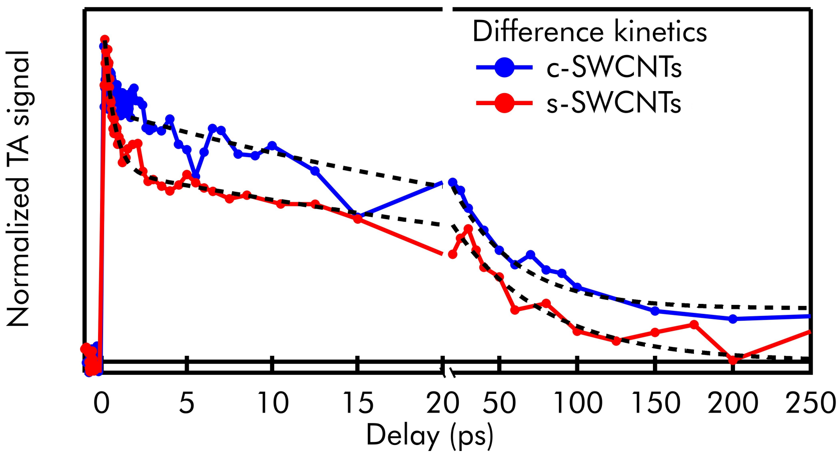

Figure S8: Difference kinetics (CD-CNT) - (bare-CD), between the TA trace at 440 nm detected in CD-CNT nanocomposites and the TA trace measured in bare CDs. The data are the same shown as black curves in Figure 4. As explained in the main paper, the data are least-square fitted by multi-exponential decay function to extract the time scale of back-electron transfer.

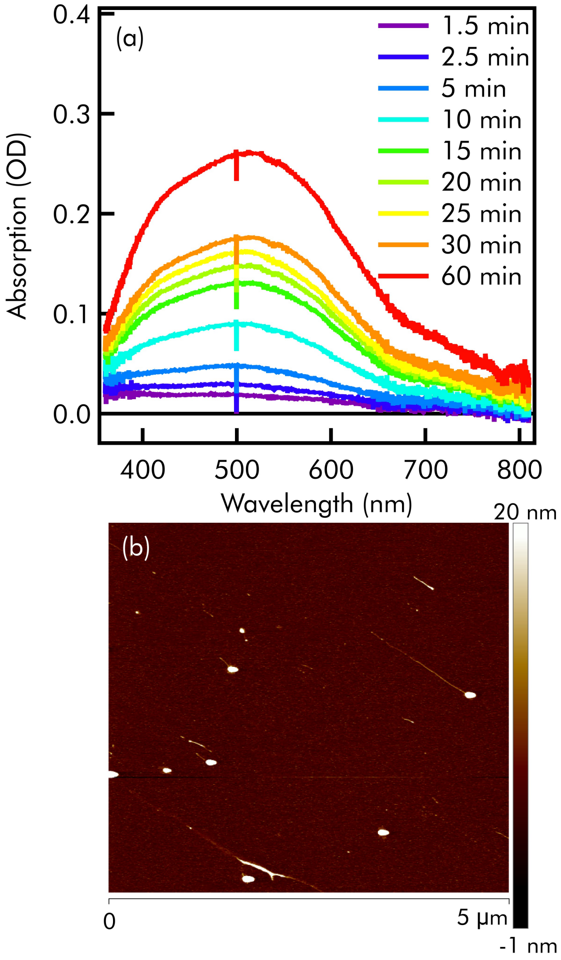

Figure S9: (a)Absorption spectra of silver nanoparticles synthesized from the light exposure of CD recorded at different exposure times. (b) AFM image of silver nanoparticles from bare CDs after 1 hour of exposure.

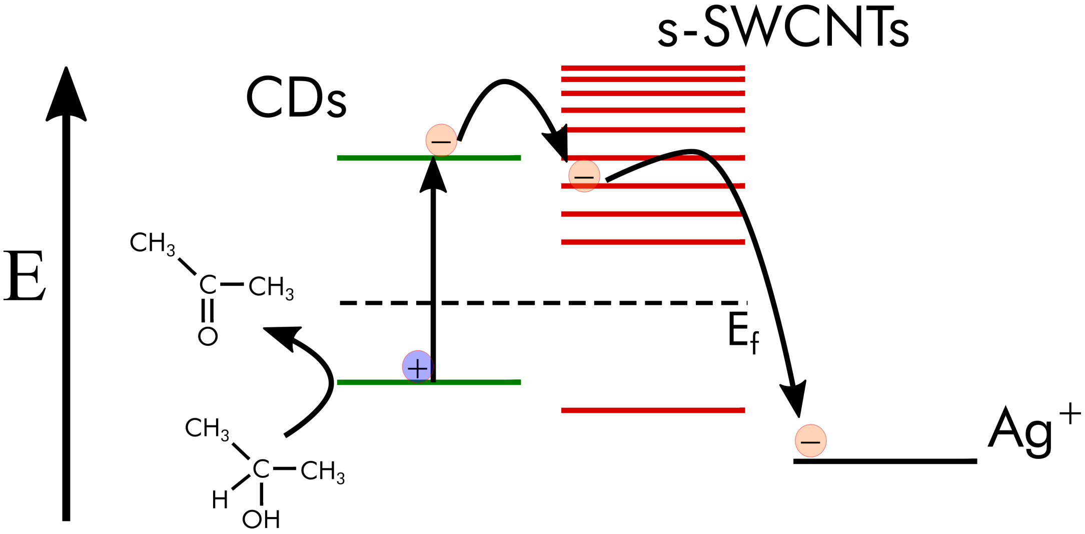

Figure S10: Schematized models of nanohybrids photocatalysis process.
